# Acquisitions and Research

**Published:** 1921-07-23

**Authors:** 


### THE COLLEGE OF SURGEONS MUSEUM.

### Acquisitions and Research.

The famous collection of specimens to be seen in the museum of the Royal College of Surgeons of England is too well known to call for any detailed introduction, but in the recently issued report of the Conservator, Prof. Sir Arthur Keith, there are several points of interest to those connected with surgery and research.

Up to 1915 the annual exhibition of recently added specimens was always fixed for the same date as the election of new members to the Council. But nowadays, when Fellows do not have to attend in person to vote, the advantage of this coincidence is lost, and therefore the exhibition will in future be held during October—namely, the opening month of the winter session of the medical schools. Also the Conservator will make the recently added specimens the subject of his autumn museum demonstrations.

### The Onodi Collection.

Among these recent additions probably the most important is a series of specimens illustrating the anatomy of the nose and its accessory sinuses, known as the Onodi Collection. Professor Adolf Onodi, of Budapest, was famous both for his researches in embryology and his writings on variations in the anatomy of this part of the body, forming thereon during his lifetime the most complete collection of illustrative preparations ever made. The acquisition by the College of this collection comes about in an unusual way. Professor Onodi died in 1919, and in the next year his son, also a medical man, brought the collection to England with a view to selling it. In face of the danger of its again going abroad, Sir St. Clair Thomson, who had previously translated certain of Professor Onodi's works, and Mr. Philip Franklin patriotically came forward and bought at their own risk the collection for £250, with the object of its being ultimately purchased and presented to the Museum. Later, the purchase-money having been raised by subscriptions, the collection was finally accepted by the College early in 1921, and it is undoubtedly a valuable acquisition.

Illustrative of the difficulties often to be overcome with such accumulations of pathological material, it is a fact that it will still cost at least £650 to mount and display the specimens of the Onodi Collection, to make no mention of the annual outlay for upkeep. In their condition as received, the preparations were quite unfit for exhibition, being still in a relatively rough state, but with all the possibilities of being made into perfect and finished exhibits. On their arrival at the College the Prosector, Mr. Henry Wilson, settled down to bring the specimens to perfection—a laborious undertaking which will occupy the greater part of two years—but, as Professor Keith claims, that the result will be commensurate with the labour involved may be seen by anyone who will visit the exhibition to be given in October next.

There is also apparently good hope that the War Office collection of pathological specimens, which is still in the upbuilding, may be ultimately entrusted to the College. To this collection alone 315 specimens have been added during the last twelve months. These include every organ and part of the body, but most notable perhaps are a series of brains and calvaria illustrating every possible variety of gunshot injury, and a series of intestines detailing the many possible lesions occurring in dysentery, typhoid, paratyphoid, bilharzia. Here, again, there is an immense amount of painstaking work to be done before one of the most professionally valuable war collections can be viewed in its entirety.

Simultaneously is proceeding the work of cataloguing and describing the Museum's marvellous accumulation of osteological specimens, which has grown round the small number of skulls and skeletons in the original Hunterian Collection. Here lies a mine of knowledge which has as yet been but partially explored. No finer collection exists in the British Empire—perhaps in the world—and it is one which can, and will, yet yield many secrets concerning the origin and relationships of the human races.

### Basis for a Complete Physical History.

In connection with this type of research, the report explains that for many years it has been the custom to accept and preserve in the Museum all human remains found in Great Britain, when they are discovered in such circumstances as to give an approximate clue to their date. In time it is hoped that sufficient material will accumulate to provide a basis for a complete physical history of the inhabitants of these islands. Professor Keith has already become convinced that definite changes, particularly in the face and jaws, have been taking place in a large proportion of the British people even during the last century or two. Only a systematic examination of ample material will decide these points, but gradually the work is being done. A record has been kept of all human remains brought to the Museum, even if the owners did not see their way to add them to the collection, and during the past year reports have, among others, described a skull from a Saxon grave, two series representing late neolithic or early bronze-age burials, neolithic and late Celtic remains from the Mendip caves. One remarkable examination has been carried out upon an entire burial from Trinidad, still embedded in its earth matrix. This example was found in an ancient shell mound, and from the pottery accompanying the remains it is inferred that the people represented belong to a period preceding the discovery of America. Year by year these specimens are dealt with, adding each time a fragment, great or small, to man's known history. Fascinating, indeed, are the multiple researches of the College of Surgeons Museum.

